# Perturbation of Interaction Networks for Application to Cancer Therapy

**Published:** 2007-04-01

**Authors:** Adrian P. Quayle, Asim S. Siddiqui, Steven J. M. Jones

**Affiliations:** Genome Sciences Centre, BC Cancer Agency, Vancouver, BC, Canada

**Keywords:** Networks, cancer, network perturbation, protein-protein interactions, drug combinations

## Abstract

We present a computational approach for studying the effect of potential drug combinations on the protein networks associated with tumor cells. The majority of therapeutics are designed to target single proteins, yet most diseased states are characterized by a combination of many interacting genes and proteins. Using the topology of protein-protein interaction networks, our methods can explicitly model the possible synergistic effect of targeting multiple proteins using drug combinations in different cancer types.

The methodology can be conceptually split into two distinct stages. Firstly, we integrate protein interaction and gene expression data to develop network representations of different tissue types and cancer types. Secondly, we model network perturbations to search for target combinations which cause significant damage to a relevant cancer network but only minimal damage to an equivalent normal network. We have developed sets of predicted target and drug combinations for multiple cancer types, which are validated using known cancer and drug associations, and are currently in experimental testing for prostate cancer. Our methods also revealed significant bias in curated interaction data sources towards targets with associations compared with high-throughput data sources from model organisms. The approach developed can potentially be applied to many other diseased cell types.

## 1. Introduction

### 1.1. Aims and motivation

Research into the genetic basis of many different diseases has developed our knowledge of the links between particular genes, proteins and diseases, such as the wellknown *P53* gene and cancer. Most diseased states are polygenic, however, and cannot be explained or characterized by a single gene, but rather by a combination of interacting genes and their products (Vogelstein et al. 2000). Therapeutic drugs traditionally target single highly connected proteins in the networks associated with diseased cells, in order to elicit a response. Some degree of efficacy is possible using such an approach, but a knowledge and characterization of the gene and protein interaction networks associated with a diseased cell state is important for the development of improved therapeutics. For example, highly connected proteins are more likely to have a critical role in the protein interaction networks of normal cells, and hence side effects and chemotoxicity will often result with such an approach (Keyomarsi and Pardee, 2003). In addition to disease therapy, a network level approach is also required to understand many other phenotypic processes, such as development (Gilbert, 2000).

We present an approach which characterizes cancer types on a network level, by developing a model of the interaction networks present in tumor cells. The primary motivation for using such an aproach is to search for potential combinations of drugs which give improved efficacy for cancer treatments compared to existing therapies typically involving a single drug. The potential improved efficacy from using a multi-drug approach to cancer therapy has already been recognized (Mitchell, 2003), but research has not yet attempted to discover novel drug combinations from a knowledge of the underlying associated networks. For example, if a given drug is known to inhibit tumor growth to some extent, we can computationally search for another drug to be used in combination with the known drug to give improved efficacy. Part of the premise of such an approach is that network topology is a useful predictor for cancer and diseased targets in general. Network topology has been shown to influence many biological properties including gene essentiality (Jeong et al. 2001; Yu et al. 2004), expression (Lukashin et al. 2003; Herrgard et al. 2003) and function (Dunn et al. 2005; Guimerà and Amaral, 2005) amongst others, and therefore some influence or correlation with target suitability seems likely. Yet, to our knowledge this correlation has not yet been studied in any detail.

We develop models of the networks associated with tumor cells and equivalent normal cells in *H. sapiens* by mapping gene expression comparison data onto networks of protein-protein interactions. The interaction data is collated both from established sources of curated *H. sapiens* interaction data and from a set of ortholog interactions in model organisms. We search for potential drug combinations using a computational simulation of the effect of removing target protein combinations from the network associated with a given tumor cell (cancer network). A number of alternative measures or ranking methods are introduced to model the criticality of these protein combinations, which are used to predict potential drug combinations by relating the relevant proteins to known drugs. A network level model is a relatively new approach to cancer research but—together with the integration of gene expression data—has already been noted as a useful avenue for drug discovery for cancer therapies (Huang, 1999). Due to current limitations in data coverage and accuracy, in particular for protein interaction data, such a network-level model cannot be expected to provide a complete solution, but may provide significant predictive power.

Interaction network models can be developed on a number of levels, using a global network model such as in the current work, possibly incorporating different network types, or alternatively using more detailed models of sub-networks or individual pathways. One of the ultimate goals of biological network models is to produce an accurate model of the complete network of interactions in a cell or tissue type, often referred to as the interactome (Walhout et al. 2002). In this approach, signalling networks, metabolic networks, and protein networks are all explicitly included in a single model, which is currently only feasible for relatively simple organisms such as *E. coli* (Juty et al. 2001). Models for more complex organisms focus on particular network types, such as transcriptional networks (Bolouri and Davidson, 2002), or metabolic networks (Kell, 2004; Stelling et al. 2002). Topological network models have shown that many cellular networks exhibit a power-law degree disribution (Jeong et al. 2000), and that their structure has a modular nature (Ravasz et al. 2002; Rives and Galitski, 2003).

### 1.2. Network perturbation

We use network perturbation to search for novel target combinations, and consider the perturbation of one network relative to another, which we call “preferential perturbation”. A single network perturbation approach attempts to maximize the perturbation to a cancer network, whereas an approach involving preferential perturbation in this case attempts to maximize the perturbation to a cancer network, while minimizing the resultant perturbation to a related normal network.

The susceptibility of networks to attack and failure has been studied for many network models and real world networks (Holme et al. 2002). The robustness of real-world networks is critical in a wide-range of contexts, including power-grids and general transport networks, communication networks and the Internet (Holme et al. 2002) (Cohen et al. 2000). Most studies have investigated the robustness of single networks or systems of networks, and have not considered the robustness of one network relative to another. The preferential perturbation of a pair of networks with non-zero similarity is a very different problem to single network perturbation, and we study the principles and topological dependency of preferential network perturbation in a separate publication (Quayle et al. 2006). We show that the extent of preferential perturbation of a pair of networks depends on a number of topological parameters, including the network similarity, size and average degree amongst others. The work in (Quayle et al. 2006) uses random (ER model) networks (Erdös and Rényi, 1960) and so called “scale-free” (BA model) networks (Barabási and Albert, 1999) to develop results and principles for general networks.

## 2. Model and Methods

We describe the methods and approach used in detail in Sections 2.1 to 2.3, in three conceptually distinct and sequential phases. The first phase is the collation of interaction data (Section 2.1), which generates underlying network models. Gene expression data is used to generate networks associated with specific cell types (Section 2.2) and the final phase involves the simulation of network perturbations to study these networks (Section 2.3). We also describe the disease and drug association data used (Section 2.4) and details of statistical techniques used for data analysis (Section 2.5).

### 2.1. Protein-protein interaction data

We combine a number of interaction data sources to improve coverage over that which could be acheived using only one such data source. We collated and populated curated *H. sapiens* interaction data from BIND (Bader et al. 2003), HPRD (Peri et al. 2003) and DIP (Xenarios et al. 2002) to give a total of 9,020 interactions between 4,524 HUGO genes (Wain et al. 2004). Since the interaction data currently available for *H. sapiens* is relatively limited in terms of coverage, we also added a set of predicted interactions in *H. sapiens* which was derived from interactions in model organisms by Lehner and Fraser (Lehner and Fraser, 2004). By mapping proteins referenced in interaction data from the model organisms *C. elegans, D. melanogaster* and *S. cerevisiae* to orthologous proteins in *H. sapiens* using the Inparanoid algorithm (O’Brien et al. 2005), a set of predicted interactions in *H. sapiens* was obtained. We extracted the core dataset of interactions which have the highest associated confidence score according to the Inparanoid mapping algorithm, to give 6,958 unique interactions. The majority of results presented here are based only on the higher confidence curated data sources, but the set of predicted interactions gives improved coverage.

### 2.2. Generation of networks associated with cell types

The combination of data sources described in Section 2.1 generates a network which is a subset of the true network of all possible interactions due to data limitations, and is also subject to the accuracy of interaction data. The particular proteins and interactions present in a cell varies significantly between cell types, depending on the gene and protein expression. A knowledge of the gene expression profile for a given cell type can be used to generate a model of the associated interaction network. We used expression comparisons of SAGE libraries (Blackshaw et al. 2003) from a particular type of tumor cell and equivalent normal cell to determine those genes which are up-regulated or down-regulated, according to a significance level of 5% and at least a five-fold change in the expression level (after conversion to a parts per million measure).

A tag to gene mapping stage is also required, for which we used the mappings available from SAGE Genie (Boon et al. 2002). Approximately 80% of the tags were successfully mapped to genes, which is a reasonable fraction for tag mapping. A more detailed discussion of alternative approaches and complications involved in tag to gene mapping is described in Pleasance and Jones (Pleasance and Jones, 2005). We map genes that are up-regulated in the cancer library to the cancer network, and down-regulated genes to the normal network, and the non-differentially regulated genes are mapped to both networks. This second phase gives two sub-networks from an underlying network to represent the networks associated with particular types of tumor and normal cells. In this phase of the method, approximately 50% of the genes map to the underlying interaction network (see Section 3.2 for more detailed discussion on data coverage).

Networks for seven different cancer types are generated from expression comparisons of short SAGE libraries available from the Cancer Genome Anatomy Project (CGAP) (Lash et al. 2000), as listed in Table 1.

### 2.3. Network perturbation

In the current work we use a topological characterization of the networks generated from the techniques described in Sections 2.1 and 2.2, as the first phase towards the development of a more detailed understanding and model of these networks. We study the change in network topology when a combination of proteins (network vertices, which we use to refer to proteins later in the paper) is removed, which we call a network perturbation. We search for optimum target combinations which, when removed, maximize the fragmentation of a given cancer network, while minimizing the fragmentation of the associated normal network. It is hoped that this may lead to novel drug combinations, which can target particular proteins, and selectively perturb the cancer network.

We measure network perturbation by the size of the giant component, which is the largest component in a network. This is a standard approach used in network robustness studies (Holme et al. 2002; Albert et al. 2000). We define the perturbation score for a single network as,

(1) $$P_{1}=\frac{G_{1b}-G_{1a}}{G_{1b}},$$

where *G*_1_*_b_* and *G*_1_*_a_* are the giant component sizes of network 1 before and after perturbation respectively (Quayle et al. 2006). Equation 1 includes normalization based on the initial network, such that the perturbation score ranges between zero and one. We define a preferential perturbation as the difference in perturbation scores for two networks such that,

(2) $$P_{12}=\frac{G_{1b}-G_{1a}}{G_{1b}}-\frac{G_{2b}-G_{2a}}{G_{2b}},$$

where *P*_12_ reflects a preferential perturbation between two networks.

An exhaustive search for the optimum vertex combination which maximizes the perturbation score is not possible due to the size of the associated search space, and a number of alternative optimization approaches were considered. It was found that a technique using successive vertex removal gives the highest score on average (Quayle et al. 2006). That is, the highest ranking vertex is initially removed, parameters are recalculated based on the new networks, the new highest ranking vertex is removed, and this process is repeated iteratively until the required number of vertices have been removed to make up a vertex combination.

Alternative vertex ranking methods were studied, based on parameters such as vertex degree, diffierence in degree, betweenness, diffierence in betweenness, and the resulting perturbation score (see (Quayle et al. 2006) for further explanation of these methods and a detailed study of their success). It was found that no single method is a universally optimal method, but rather the best method depends on the regime of interest (number or fraction of vertices to remove) and also details of network topology. Therefore we present and compare results from all methods considered for the perturbation of cancer and normal networks.

### 2.4. Target association data

Given a novel target combination we wish to determine whether those targets are druggable or disease associated, and in particular whether they are cancer associated. We used the Therapeutic Target Database (TTD) as a source of target-drug associations (Chen et al. 2002), which includes information on protein and nucleic acid targets, the diseased target, corresponding drugs or ligands and pathway information. After mapping to HUGO IDs we obtained 659 targets, 231 of which have drug associations, and 641 with disease associations at the time of population. Many of the listed disease associations are cancer associations, and many targets are associated with multiple cancers. An additional set of 299 cancer associations was obtained from a literature census of mutated genes implicated in human cancer (Futreal et al. 2004), giving 275 associations after mapping to HUGO IDs.

### 2.5. Correlation tests

We studied the correlation of vertex properties between networks which provides a number of insights, but is principally useful for quantifying the influence of the underlying network on the resulting cancer and normal networks. The types of analysis are based either on initial network topology or perturbation results, for which we use standard linear regression (Section 2.5.1) or Spearman’s rank correlation coefficient (Section 2.5.2) respectively.

#### 2.5.1. Initial topology

Correlations between vertex topological properties have been studied for model networks (Holme et al. 2002; Quayle et al. 2006), and a similar analysis can be made for the cancer and normal networks, and the underlying network. For example, the correlation between vertex degree and betweenness for a given network is visualized by a scatter plot, where the *x* and *y* values of each point are the degree and betweenness of a vertex. A least-squares linear regression coefficient is used to quantify the strength of the correlation. We calculate the linear correlation coefficients between different cancer networks and between the underlying network and cancer networks.

#### 2.5.2. Perturbation results

The output from a perturbation simulation is an ordered vertex list, that is, a set of vertex ranks. The correlation between the rank positions of vertices in two different perturbations can be calculated using Spearman’s rank correlation coefficient which is suitable specifically for ranked data. The output from two perturbations typically contains different vertex sets and a different number of vertices, so the coefficient is calculated based only on vertices in both output lists. Spearman’s rank correlation coefficient, *r_s_* is given by the expression,

(3) $$r_{s}=1-\frac{6\sum_{i=1}^{n}d_{i}^{2}}{n^{3}-n},$$

where *n* is the number of paired ranks, and *d_i_* is the diffierence between the two ranks in a pair (in this case for a given vertex) (Zar, 1999). Note that the above expression is strictly only valid for data with no tied ranks, which is true for the perturbation results since only one vertex is removed at a time.

## 3. Results and Discussion

### 3.1. Protein-protein interaction data

The underlying network model constructed from the combination of data sources was analyzed by calculating a range of network properties. Table 2 shows relevant statistics for the data sources and data source combinations, where we use the network terminology vertices and edges to refer to proteins and protein-protein interactions respectively.

A number of network parameters are shown in Table 2 which we explain in more detail below, since these parameters are discussed in later sections of this paper. The degree of a vertex is the number of edges connected to that vertex, and the average degree is the vertex degree averaged across all vertices in a network. Similarly, there is a shortest path length between every pair of vertices in a network, and the path length is averaged over all vertex pairs to give the average shortest path. The betweenness of a vertex (Joy et al. 2005), which is discussed later, measures the number of shortest paths passing through a given vertex.

The clustering coefficient measures the fraction of transitive triples or triangles between nearest neighbours, and the clustering coefficient of vertex *i*, *C_i_*, is given by,

(4) $$C_{i}=\frac{2E_{i}}{k_{i}\left(k_{i}-1\right)},$$

where *k_i_* is the degree or number of nearest neighbours of vertex *i*, and *E_i_* is the number of edges connecting between these nearest neighbours (Watts and Strogatz, 1998; Wasserman and Faust, 1994). The clustering coefficient of a network is calculated by averaging *C_i_* over all vertices of the network. Finally the assortativity coefficient measures the tendency of vertices to connect to other vertices which have a similar degree (see (Newman, 2002) for an exact definition). In other words, if the “hubs” in a network tend to connect to other hubs, then the network shows assortative mixing. The assortativity coefficient is defined between minus one for a perfectly disassortative network and plus one for a perfectly assortative network, where a value of zero means there is no assortative mixing. It has been shown that social networks are generally assortative, whereas biological and technological networks are generally disassortative (Newman, 2002).

We group the data sources into two data source combinations, where the first is a combination of primary *H. sapiens* interaction data, and the second combination contains all data sources including predicted interactions. DIP has the least interactions and the smallest average degree, and correspondingly the largest average shortest path. The data source of predicted interactions is the most connected, and has a surprisingly high assortativity coefficient of *r* = 0.446, since biological networks are generally disassortative. This likely reflects sample bias in this dataset of core interactions, since each data source is only a subset of the complete set of interactions, and is subject to bias in terms of known interactions. This bias and the fact that data derived from orthologous protein-protein interactions is expected to be less accurate than primary *H. sapiens* interactions are the motivation for using two alternative data source combinations.

### 3.2. Topology and similarity of cancer and normal network pairs

We use the definition of network similarity, *S*, in terms of network edges given by,

(5) $$S=\frac{n_{c}}{n_{t}},$$

where *n_c_* is the number of edges in common between the two networks, and *n_t_* is the total number of edges in the combined network (see (Quayle et al. 2006) for further explanation). At the two extremes, if the networks are identical then no preferential perturbation is possible, but if the networks have no similarity then a complete perturbation of one network relative to the other is possible if sufficient vertices are removed, and the problem is equivalent to single network perturbation. Between these limits, some maximum possible preferential perturbation score is associated with two given networks.

A detailed derivation of the similarity of both independent and “correlated” network pairs has been determined (Quayle et al. 2006). Correlated networks are derived from a single underlying network, and the cancer and normal network pairs are correlated network pairs, since they are derived from the underlying interaction network. The similarity of such correlated networks is independent of network topology if the vertices which make up the correlated networks are sampled randomly from the underlying network. The similarity of randomly sampled correlated networks varies directly with the vertex set similarity, *V_S_*, which is given by,

(6) $$V_{S}=\frac{N_{C}}{N_{T}},$$

where *N_C_* is the number of vertices common to the two networks, and *N_T_* is the total number of distint vertices in the networks. For random vertex sampling, we showed by analytical derivation that the expected network similarity, *E*(*S*), is equal to the square of the vertex set similarity.

(7) $$E(S)\;=V_{S}^{2}$$

**Figure 1.**
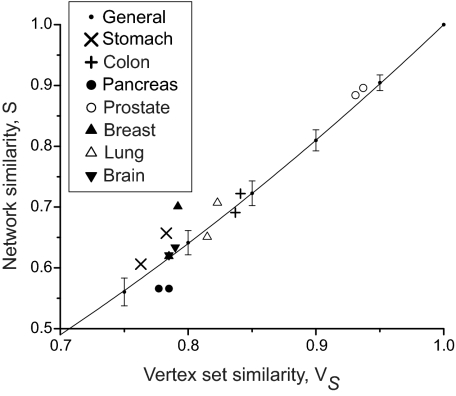
Network similarity and vertex set similarity values for cancer and normal network pairs (data combinations C1 and C2), compared directly to the expected network similarity for a general network topology with random vertex sampling.

Fourteen cancer and normal network pairs were generated from the seven cancer types listed in Section 2.2 and the two data source combinations described in Section 3.1. After tag mapping, the results from an expression comparison contain around 7,000 genes, which is typically reduced to around 2,000 after singletons—a SAGE tag with only one occurrence in the corresponding library—are removed. There is significant variation in this stage, however, and some cancer types such as prostate have far fewer singletons, leaving around 4,000 genes after the removal of singletons. Approximately 50% of these genes map to the underlying network, giving network sizes of the order of 1,000 genes. The similarity of the generated network pairs was calculated and plotted against the vertex set similarity for each network pair, as shown in Figure 1. The variation of the expected network similarity for a general network pair with random vertex sampling is also shown.

Figure 1 shows that the network similarity and vertex set similarity values for the cancer and normal networks are generally close to the predicted variation for random vertex sampling. Network pairs for different cancer types have network similarity values within the approximate range 0.55 ≤ *S* ≤ 0.70, with the exception of prostate cancer, where *S* ∼ 0.90. Therefore the maximum preferential perturbation score is likely to be lower for the prostate cancer networks, depending also on other topological parameters such as network-size, average degree and clustering coefficient, which all influence network robustness to some extent (Quayle et al. 2006). Table 3 show the values of such parameters for selected networks, which is a sample from a larger table in the Supplementary Materials section.

### 3.3. Preferential perturbation of cancer and normal network pairs

#### 3.3.1. Vertex ranking methods

**Figure 2.**
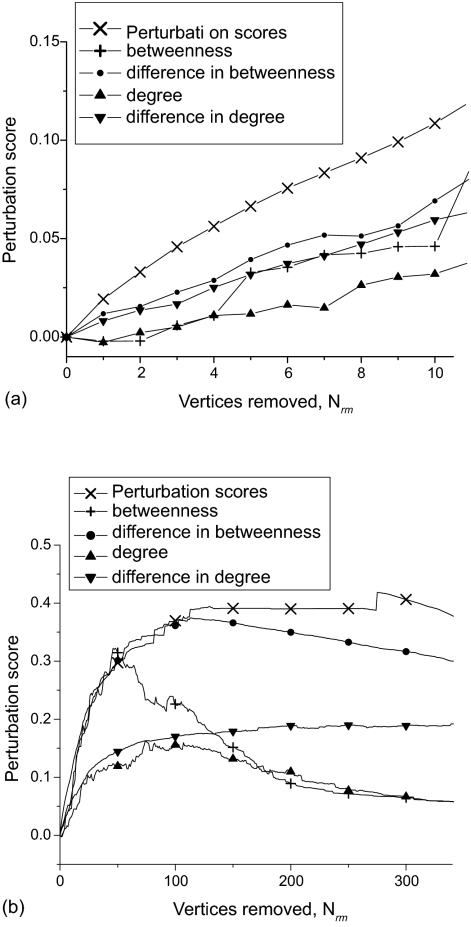
Preferential perturbation averaged over fourteen cancer and normal network pairs, for alternative vertex ranking methods and for initial vertex removals (small *N_rm_*) (**a**), and large *N_rm_* up to maximum (**b**).

We study the preferential perturbation of cancer and normal networks using the vertex ranking methods described in Section 2.3. We are primarily interested in removing combinations of only a few vertices (proteins) to give novel target combinations, which corresponds to a small fraction of the total number of vertices in a given network. Successive vertex removal is applied until a network is fully fragmented and all vertices are removed, which provides useful additional network characterization. We refer to the variation of the perturbation score with the number of vertices removed as a “perturbation profile”. Figure 2 shows preferential perturbation profiles averaged across the fourteen cancer and normal network pairs based on the number of vertices removed. Notably a preferential perturbation profile generally increases up to a maximum, and if more vertices are removed beyond this point the score decreases, as the normal network becomes more fragmented.

As predicted from results developed in (Quayle et al. 2006), Figure 2 shows that no single vertex ranking method is universally optimal, but rather the best method depends on the regime of interest and the definition or metric of method success. Alternative metrics of success are discussed further below, but often the extent of perturbation or perturbation score may be the most suitable metric. According to this metric, for small *N_rm_* the perturbation scores method is the most effective, and betweenness-based methods are generally more effective than degree-based methods.

For ER and BA model networks degree-based methods are in fact more effective than betweenness-based methods in this regime. The cancer and normal networks are significantly more clustered than equivalent ER and BA model networks of the same size and average degree, as shown in Table 4, which is partly responsible for the observed difference in effectiveness of these methods. The observed clustering fits results from previous studies which have shown that many biological networks have an inherent modularity and clustering (Ravasz et al. 2002; Girvan and Newman, 2002).

The success of vertex ranking methods relates closely to whether or not they provide an accurate measure of the “centrality” of vertices in a network. As discussed, degree and betweenness are alternative measures of vertex centrality, and for most network topologies betweenness-based methods are more effective (Holme et al. 2002; Quayle et al. 2006). This is not surprising, since betweenness is a global measure (requires knowledge of network structure) whereas degree is only a local measure (detailed network structure is not needed to measure the degree of a given vertex). The perturbation scores method is a relatively poor measure of vertex centrality, since this method effectively forces at least some perturbation for each vertex removal, and hence tends to target more peripheral vertices. Therefore, although this method on average gives the greatest preferential perturbation score for small *N_rm_*, this may not be the most effective predictor of useful targets.

Alternative metrics for analyzing method effectiveness are the score at the maximum in a profile, and the average perturbation gradient, which is given by,

(8) $$grad(P_{12})=\left(\frac{P_{12}}{N_{rm}/N}\right)max,$$

where *max* represents the values of these parameters at the maximum in a given profile (see (Quayle et al. 2006) for the motivation behind these parameters). When searching for novel target combinations, we focus on combinations of up to 5 targets, in other words the regime for small *N_rm_*, below the maximum. The current static topological model gives a conservative estimate of the fragmentation of a real network, since it cannot take into account dynamical effects such as possible cascades and subtle dependencies between interactions, which may be observed in the real biological network. Therefore, alternative metrics based on the maximum in a profile may be more realistic, or at least equally effective predictors of the effect of perturbations on a network, rather than simply the score at a given *N_rm_* value.

Tables 5 and 6 show values for these metrics averaged across the fourteen network pairs for each vertex ranking method. Vertex betweenness is significantly the most effective method according to both metrics, which cannot be seen in the averaged profiles shown in Figure 2 since maxima occur at different *N_rm_* values in different networks. These results show a similar order of method effectiveness as predicted (Quayle et al. 2006), and suggest that the betweenness method may provide the most useful target predictions.

Target results for prostate cancer networks for combinations of up to 5 targets are shown in Table 7, for which we are testing the predictions in our own prostate cancer research group. Although betweenness is the most effective method according to the two metrics as shown in Tables 5 and 6, the first few vertex removals using this method actually give a negative perturbation score for this particular network pair (i.e. the normal network is more fragmented than the cancer network). On the other hand, diffierence in betweenness generates a comparable score to that generated using the perturbation scores method.

The results shown in Figure 2 and Tables 5 to 7 give strong evidence that degree-based methods are less useful than betweenness-based methods. Furthermore, betweenness and difference in betweenness are more effective measures of vertex centrality than degree and difference in degree respectively. What is less clear from the above analysis is exactly which metric of method success is most appropriate, given the current model. Therefore we are initially investigating highly ranked targets from the betweenness, diffierence in betweenness and perturbation scores in experimental testing.

#### 3.3.2. Correlation tests

Targets with a high betweenness in a given cancer network also tend to have a high betweenness in the underlying network from which the cancer network was derived. This has the result that highly ranked targets according to the betweenness method for a given cancer type are often highly ranked targets in another cancer type, due to the correlation of both cancer networks with the underlying network. The difference in betweenness ranking method selects targets which are less strongly correlated with the topology of the underlying network, and thus the highly ranked targets tend to be more specific to a given cancer type. It is possible that both approaches may predict useful novel targets, since some known cancer targets are highly specific to a given cancer type (Fukazawa et al. 2004), while others are known to provide a therapeutic response in many cancer types (Shelton et al. 2005).

To analyze the strength of these correlations between the underlying network and the cancer and normal networks, and between different cancer types, we used correlation tests as described in detail in Section 2.5.1. Table 8 shows the vertex betweenness correlation between the underlying networks and cancer networks. Typical correlation coefficients are within the approximate range 0.8 < *r* < 0.9, indicating a significant correlation in this case. Correlations between vertex degree are slightly stronger between a cancer network and the underlying network from which it was derived, but weaker between a cancer network and a different underlying network (see Supplementary Materials). This is an interesting result which shows that betweenness is less sensitive to the addition or removal of links (between different underlying networks) than degree, and that vertex betweenness captures the overall or core topology of a network more effectively than vertex degree. This inference is consistent with the fact that betweenness provides a better measure of vertex centrality than degree. Since the current underlying networks are only subnetworks of the true or complete interaction network, this result indicates that such a subnetwork can provide a useful representation of the “core” topology of the true interaction network.

It is expected that the observed correlations with initial network topology extend to the vertices (targets) selected in perturbations in different cancer types, since perturbation ranking methods are based on betweenness and degree. We investigated the vertex correlations between perturbation results for different cancer types using Spearman’s rank correlation coefficient, as described in detail in Section 2.5.2. Correlation coefficients were calculated between each cancer network or cancer and normal network pair, for each vertex ranking method. The Supplementary Materials contains detailed results for the betweenness and difference in betweenness ranking methods, and Table 9 shows the average rank correlation coefficient for each vertex ranking method.

The average correlation coefficients for the betweenness and degree ranking methods are significantly higher than those from other methods, since these methods do not take into account the relevant normal network topology. Therefore a target with a high rank in the perturbation results using the betweenness method in a given cancer type, is likely to also have a high rank in the results for another cancer type, if it is present in the network (recall the correlations are only based on common targets). There is less correlation in the perturbation results using other ranking methods, and the diffierence in betweenness ranking method gives the least correlation, reflecting the sensitivity of this method to topological differences. Correlations between perturbation results were also compared between the underlying network and the cancer networks, giving similar results for different ranking methods.

#### 3.3.3. Target associations

The outcome of the current work is a set of ranked targets for a given cancer type, which we are testing for their potential as targets for cancer therapy. An approach for assessing the likelihood that these targets are useful, potentially novel cancer targets is to validate the target ranks against known cancer targets. If our methods preferentially select for known cancer targets, this indicates that highly ranked novel targets have a greater likelihood of being useful cancer targets. We applied a global statistical analysis on the ranks of targets with known cancer associations, disease associations and drug associations, using the association data described in Section 2.4. The null hypothesis is that targets with a specified association type are selected randomly from a given network, and the alternative hypothesis is that such targets have a greater chance of selection by our methods, or in other words are more highly ranked than at random. The analysis generates a p-value using hypergeometric probability distributions for each cancer and normal network pair for a given association type and ranking method. These p-values are then combined using the Z-transform test (Whitlock, 2005) to give an overall p-value for each ranking method, for a given association type. The combined p-value therefore measures the ability of a given ranking method to preferentially select for cancer targets, for example.

The resulting p-values are given in Table 10, which shows that according to the null hypothesis the betweenness, degree and perturbation scoring methods all strongly select for cancer and disease associated targets. This seems to validate the effectiveness of these methods, but the null hypothesis is naive, since it assumes independence between tests and does not account for possible biases or network correlations as calculated in Section 3.3.2. As discussed, we expect some biases in the underlying interaction data for known cancer targets, since these targets are known to be therapeutically interesting. These biases will tend to increase the degree and betweenness of targets with associations in the underlying networks and cancer and normal networks. We therefore calculate the average degree and betweenness of targets with associations in these networks, and compare them with the average degree and betweenness of all targets in the networks. Tables 11 and 12 show ratio values for the average betweenness for different association types.

The ratio values are consistently greater than 1, which shows that targets with associations have significantly greater betweenness than an average target, both in the cancer networks and the underlying networks. The average value of the equivalent ratios for the average degree are typically around 1.5 (see Supplementary Materials), which is less than the ratios for average betweenness. The betweenness distributions of these networks have a larger spread or standard deviation than that of equivalent degree distributions, which in part explains this difference.

Since targets with associations tend to have a higher than average betweenness and degree in the underlying curated networks, much of the significance observed in Table 10 is due to the influence of the underlying network, rather than the cancer specific networks. This result is biologically reasonable, and is potentially very useful for the discovery of cancer targets with therapeutic applications in many cancer types. For example, the well-characterized EGFR target is highly ranked from our methods in many cancer types, and is known to be implicated in many cancer types including prostate cancer (Shelton et al. 2005), where it is the fifth highest ranked target using the betweenness ranking method. Many other key cancer targets are highly-ranked using our methods in different cancer types, such as SMAD3, MAPK3, RAF1 and TP53, amongst others.

In order to quantify the bias in these curated datasets to some extent, we ran a similar analysis using networks generated only from high-throughput data. All methods of network generation and analysis are as described previously except the underlying network is now the complete set of interactions predicted by Lehner & Fraser (Lehner and Fraser, 2004). This high throughput data is biased, but the types of bias are different to those expected in curated data, and therefore such an analysis is useful (Mrowka et al. 2001; Serebriiskii et al. 2000). Tables 13 and 14 show equivalent results for networks generated from high-throughput data to the results in Tables 11 and 12 for curated interaction data (see Supplementary Materials for degree ratios).

The betweenness ratios are much lower for networks generated from high-throughput data than from curated data, which indicates that much of the significance is likely to be due to biases in the data, and gives a clear demonstration of bias in curated interaction data sources. Interestingly, the betweenness and degree ratios are still significant for targets with drug associations, which shows how drugs have typically been developed for relatively “central” targets with high degree and betweenness. We also ran perturbations of networks generated from high-throughput data, and applied the same analysis as described for curated data sources to generate Table 10. Equivalent results from high-throughput data sources are shown in Table 15. In this case, none of the association types are significantly selected, with the exception of drug associations, which are selected by the betweenness and degree methods. These results are consistent with the differences between network properties of targets with associations for curated and high-throughput data as shown in Tables 11 to 14.

The differences in results for curated and high-throughput interaction data sources highlight some of the current problems with interaction data, where curated sources contain significant biases towards well studied targets of interest, and high-throughput sources contain a high percentage of false positives. As more high-quality interaction data becomes available, a network-level approach will become increasingly important for target and drug discovery. Given the current data limitations, however, our predictions for novel target combinations have returned many targets known to be important in the relevant cancer types, and we are pursuing these predictions for their applicability in cancer therapy. We have also run perturbations where only targets with known drug associations are selected so that the results can be tested using readily available drug combinations. Many of the highest ranked targets with drug associations are also known cancer targets.

## 4. Summary

In summary, we have developed and studied a novel method for predicting target and drug combinations based on network topology in multiple cancer types. By simulating network fragmentation from targeting multiple proteins in a given cancer type, such a network-level approach facilitates a search for novel target combinations. Our methods significantly select for cancer associated targets using curated interaction data sources, and return many targets of interest in cancer therapy. When using predicted *H. sapiens* interaction data generated from high-throughput model organism data sets, the methods do not significantly select for known cancer associations. The difference in results between networks generated from curated data sources and high-throughput data sources reveals significant bias in curated data towards targets of interest, with known associations. We have predicted sets of target and drug combinations in seven different cancer types, which are currently in experimental testing for prostate cancer using both drug studies and siRNA techniques.

## Supplementary Material

**Table 1.** Target lists for stomach cancer and networks of data combination C1.

Vertices removed	betweenness	difference in betweenness	degree	difference in degree	perturbation scores
1	PXN	G22P1	PXN	PXN	GAPD
2	VCL	VCL	VCL	VCL	CTBP1
3	EGFR	COL1A1	EGFR	COL1A1	CTSB
4	SMAD3	EGFR	RNF11	APEX1	COL1A1
5	COL1A1	PXN	SMAD3	COL1A2	MIF
6	PIN1	SMAD3	RAF1	KRT18	RAF1
7	CTNNB1	UBE2I	JUN	G22P1	EGFR
8	UBE2I	SFRS1	COL1A1	HSPH1	C1QBP
9	MAPK3	HSPA1A	CDC42	CTSB	G22P1
10	HSPA1A	APEX1	HDAC2	HSPD1	APEX1

**Table 2.** Target lists for colon cancer and networks of data combination C1.

Vertices removed	betweenness	difference in betweenness	degree	difference in degree	perturbation scores
1	VCL	SMAD2	PXN	SMAD2	SMAD2
2	PXN	GNB2L1	VCL	GNB2L1	BCAP31
3	TLN1	MYC	TLN1	FUS	VCL
4	UBE2I	FUS	SMAD2	MYC	PXN
5	PCNA	HLA-C	PLK1	HLA-C	CALR
6	PIN1	RPS20	FNBP3	VCL	MAPK1
7	SMAD2	MAPK1	CDC42	SRRM1	FUS
8	GNB2L1	IDE	PCNA	SACM1L	APP
9	SMARCA4	SNRPC	APP	IGF2	PCNA
10	PLK1	RAC1	SP1	NPM1	YWHAZ

**Table 3.** Target lists for pancreas cancer and networks of data combination C1.

Vertices removed	betweenness	difference in betweenness	degree	difference in degree	perturbation scores
1	JUN	ERBB2	FNBP3	RELA	ERBB2
2	HDAC3	JUN	RELA	SMAD3	LRP1
3	SMAD3	SMAD3	ITGB1	COL1A1	NCL
4	ERBB2	RELA	SMAD3	THBS1	RELA
5	UBE2I	NCL	APP	ERBB2	MMP2
6	CSNK2A1	THBS1	JUN	FOS	SMAD3
7	GNB2L1	APP	HDAC3	EP300	FNBP3
8	ZNF265	UBE2I	COL1A1	COL1A2	GSN
9	APP	YY1	YY1	LRP1	ACTG1
10	YY1	HDAC3	ERBB2	NCL	CTSB

**Table 4.** Target lists for prostate cancer and networks of data combination C1.

Vertices removed	betweenness	difference in betweenness	degree	difference in degree	perturbation scores
1	PXN	SERPINA3	PXN	SERPINA3	APP
2	VCL	APP	VCL	SLC9A3R1	CDC42
3	SMAD2	SLC9A3R1	SMAD2	HIST2H2BE	MAPK3
4	TLN1	DSP	TLN1	DSP	CDKN1A
5	EGFR	HIST2H2BE	SMAD3	CBX4	PBX2
6	SMAD3	YWHAZ	RNF11	KRT18	HSPH1
7	CREBBP	IQGAP2	EGFR	COPS3	COL4A1
8	SRC	MAPK3	CREBBP	CCNB1	HIST2H2BE
9	HDAC3	COPS3	RAF1	TOMM20	VDAC1
10	RAF1	DCN	EP300	RPS3KA1	SHC1

**Table 5.** Target lists for breast cancer and networks of data combination C1.

Vertices removed	betweenness	difference in betweenness	degree	difference in degree	perturbation scores
1	VCL	SMAD2	PXN	SMAD2	SMAD2
2	PXN	COL1A1	VCL	COL1A1	EP300
3	TLN1	ERBB2	TLN1	COL1A2	MPHOSPH6
4	SMAD3	COL1A2	SMAD2	LMO4	NFKB1
5	SMAD2	CSK	SMAD3	ERBB2	PTMA
6	ERBB2	RNF11	FNBP3	A2M	NFKBIB
7	PAK1	CCT3	CREBBP	CSK	PBX2
8	FNBP3	LGALS1	ITGB1	EXOSC4	PXN
9	XPO1	YY1	RNF11	PBX2	UBE2I
10	PTMA	RELB	RAF1	SLC9A3R1	VCL

**Table 6.** Target lists for lung cancer and networks of data combination C1.

Vertices removed	betweenness	difference in betweenness	degree	difference in degree	perturbation scores
1	VCL	YWHAH	VCL	PCNA	BECN1
2	SMAD3	PCNA	SMAD3	LRP1	NFKB2
3	JUN	LRP1	PCNA	A2M	CTNNB1
4	CSNK2A2	DDX3X	RNF11	HLA-DRA	YWHAH
5	FNBP3	CSNK2A2	FNBP3	CUL1	HLA-C
6	RNF11	AKT1	NFKBIA	HLA-C	LRP1
7	HSF1	CD63	JUN	COL1A2	CD63
8	HDAC3	B2M	APP	YWHAH	AUP1
9	AKT1	HSPA5	MPHOSPH6	TFRC	CSNK2A2
10	A2M	SPARC	HDAC3	CEBPA	AKT1

**Table 7.** Target lists for brain cancer and networks of data combination C1.

Vertices removed	betweenness	difference in betweenness	degree	difference in degree	perturbation scores
1	VCL	VCL	VCL	ITGB1	HLA-C
2	TP53	ITGB1	TP53	HSPA1A	ITGB1
3	TLN1	RAF1	TLN1	COL1A1	RAF1
4	SMAD3	TP53	SMAD3	RAF1	SMAD3
5	CTNNB1	PIK3R1	RNF11	VCL	HD
6	PIK3R1	HSPA1A	HD	COL1A2	RAN
7	RAF1	SMAD3	CTNNB1	NFKBIA	HSPA1A
8	RNF11	KIT	APP	XPO1	PTK2B
9	HSF1	TLN1	ITGB1	TGFBR2	ITGA5
10	HD	COL1A1	E2F4	RAN	LSM1

**Table 8.** Network properties.

Network	Number of vertices, *N*	Number of edges, *n*	Giant component size, *G*	Assortativity coefficient, *r*	Average degree, *k*	Average shortest path, *l*	Clustering coefficient *C*	Modularity, *Q*
C1 stomach cancer	887	586	414	−0.1499	1.3213	5.8250	0.0309	0.7666
C1 stomach normal	848	501	366	−0.1843	1.1816	5.7118	0.0271	0.7620
C1 colon cancer	992	709	502	−0.1325	1.4294	5.5504	0.0285	0.7332
C1 colon normal	1069	843	569	−0.1197	1.5771	5.6176	0.0343	0.7477
C1 pancreas cancer	898	585	391	0.0584	1.3028	6.8627	0.0335	0.7837
C1 pancreas normal	859	452	286	0.0106	1.0523	6.7659	0.0299	0.7806
C1 prostate cancer	1627	1462	926	−0.0992	1.7971	5.8279	0.0468	0.7166
C1 prostate normal	1654	1517	954	−0.0960	1.8343	5.8097	0.0465	0.7137
C1 breast cancer	1243	976	620	−0.1250	1.5703	5.6087	0.0464	0.7062
C1 breast normal	1293	1100	690	−0.1168	1.7014	5.4527	0.0421	0.7072
C1 lung cancer	1085	685	450	−0.1023	1.2626	6.2026	0.0374	0.7633
C1 lung normal	1120	811	574	−0.0955	1.4482	6.2918	0.0345	0.7698
C1 brain cancer	1522	1360	830	−0.0962	1.7871	5.5338	0.0427	0.6749
C1 brain normal	1457	1190	766	−0.0910	1.6334	5.8138	0.0392	0.7042
C2 stomach cancer	1158	1243	641	0.0961	2.1468	5.6723	0.0869	0.7324
C2 stomach normal	1129	1064	580	0.1300	1.8848	5.7495	0.0789	0.7507
C2 colon cancer	1300	1439	720	0.0379	2.2138	5.4863	0.0858	0.7145
C2 colon normal	1392	1565	807	0.0227	2.2485	5.5766	0.0924	0.7109
C2 pancreas cancer	1137	1072	601	0.3602	1.8856	6.6753	0.0901	0.7829
C2 pancreas normal	1119	1221	557	0.6085	2.1823	6.4320	0.1017	0.7649
C2 prostate cancer	2141	3107	1457	0.1687	2.9023	5.6441	0.0953	0.6868
C2 prostate normal	2168	3230	1488	0.1888	2.9797	5.6457	0.0946	0.6794
C2 breast cancer	1651	2066	980	0.1330	2.5027	5.5949	0.0935	0.6677
C2 breast normal	1665	2161	1008	0.1419	2.5957	5.4645	0.0885	0.6649
C2 lung cancer	1408	1593	772	0.2717	2.2627	6.1970	0.0905	0.7628
C2 lung normal	1458	1865	901	0.2637	2.5582	5.9925	0.0921	0.7318
C2 brain cancer	2016	2746	1327	0.1809	2.7242	5.6988	0.0940	0.6672
C2 brain normal	1939	2420	1222	0.1062	2.4961	5.8829	0.0861	0.7127

**Table 9.** Network similarity for every pair of cancer networks.

Network	C1 stomach	C1 colon	C1 pancreas	C1 prostate	C1 breast	C1 lung	C1 brain	C2 stomach	C2 colon	C2 pancreas	C2 prostate	C2 breast	C2 lung	C2 brain
C1 stomach	1.0000	0.2148	0.1571	0.2220	0.2290	0.1812	0.1924	0.4714	0.1275	0.1061	0.1120	0.1233	0.0983	0.1040
C1 colon		1.0000	0.1817	0.2321	0.2166	0.2007	0.1918		0.4927	0.1258	0.1200	0.1212	0.1126	0.1067
C1 pancreas			1.0000	0.1791	0.1597	0.2282	0.1911			0.5457	0.0920	0.0883	0.1215	0.1033
C1 prostate				1.0000	0.3086	0.2089	0.2845				0.4706	0.1947	0.1382	0.1744
C1 breast					1.0000	0.1975	0.2192					0.4724	0.1194	0.1272
C1 lung						1.0000	0.2158						0.4300	0.1183
C1 brain							1.0000							0.4953

C2 stomach	0.4714	0.1329	0.0953	0.1595	0.1509	0.1125	0.1372	1.0000	0.2428	0.1698	0.2299	0.2620	0.2161	0.2161
C2 colon		0.4927	0.1090	0.1641	0.1418	0.1232	0.1350		1.0000	0.1856	0.2445	0.2281	0.2320	0.2219
C2 pancreas			0.5457	0.1399	0.1173	0.1552	0.1472			1.0000	0.1835	0.1661	0.2103	0.1712
C2 prostate				0.4706	0.1639	0.1084	0.1627				1.0000	0.3370	0.2587	0.3284
C2 breast					0.4724	0.1106	0.1397					1.0000	0.2395	0.2727
C2 lung						0.4300	0.1402						1.0000	0.2580
C2 brain							0.4953							1.0000

**Table 10.** Vertex set similarity for every pair of cancer networks.

Network	C1 stomach	C1 colon	C1 pancreas	C1 prostate	C1 breast	C1 lung	C1 brain	C2 stomach	C2 colon	C2 pancreas	C2 prostate	C2 breast	C2 lung	C2 brain
C1 stomach	1.0000	0.3441	0.3164	0.3531	0.3867	0.3563	0.3458	0.7660	0.2819	0.2690	0.2766	0.3056	0.2915	0.2710
C1 colon		1.0000	0.3539	0.3748	0.3440	0.3930	0.3516		0.7631	0.3021	0.2952	0.2762	0.3230	0.2778
C1 pancreas			1.0000	0.3539	0.3216	0.3877	0.3297			0.7898	0.2774	0.2569	0.3162	0.2593
C1 prostate				1.0000	0.4576	0.4154	0.4833				0.7599	0.3790	0.3555	0.3921
C1 breast					1.0000	0.3832	0.4143					0.7529	0.3215	0.3307
C1 lung						1.0000	0.3882						0.7706	0.3073
C1 brain							1.0000							0.7550

C2 stomach	0.7660	0.2882	0.2637	0.3081	0.3287	0.3003	0.3003	1.0000	0.3294	0.3077	0.3438	0.3643	0.3449	0.3325
C2 colon		0.7631	0.2899	0.3226	0.2902	0.3257	0.3017		1.0000	0.3434	0.3622	0.3359	0.3795	0.3366
C2 pancreas			0.7898	0.3137	0.2803	0.3321	0.2914			1.0000	0.3401	0.3065	0.3661	0.3127
C2 prostate				0.7599	0.3629	0.3276	0.3891				1.0000	0.4545	0.4017	0.4783
C2 breast					0.7529	0.3085	0.3428					1.0000	0.3644	0.4082
C2 lung						0.7706	0.3312						1.0000	0.3779
C2 brain							0.7550							1.0000

**Table 11.** Linear regression correlation coefficients for initial vertex degree between underlying networks and corresponding cancer networks.

	C1	C2
C1 stomach	0.8494	0.6805
C1 colon	0.8366	0.6852
C1 pancreas	0.8215	0.6810
C1 prostate	0.9079	0.6832
C1 breast	0.8948	0.6991
C1 lung	0.8779	0.6619
C1 brain	0.9157	0.7050
C2 stomach	0.6080	0.8842
C2 colon	0.6503	0.8725
C2 pancreas	0.5718	0.8582
C2 prostate	0.6442	0.9254
C2 breast	0.6304	0.9077
C2 lung	0.5414	0.8895
C2 brain	0.6516	0.9233

**Table 12.** Comparison of perturbation results (target rankings) using Spearman’s rank correlation coefficient based on the betweenness method. Only the common targets are compared, and the ranks are relative to this target set.

Network	C1 stomach	C1 colon	C1 pancreas	C1 prostate	C1 breast	C1 lung	C1 brain	C2 stomach	C2 colon	C2 pancreas	C2 prostate	C2 breast	C2 lung	C2 brain
C1 stomach	1.0000	0.8163	0.7057	0.5739	0.7976	0.7721	0.6502	0.7413	0.5992	0.5602	0.4665	0.4389	0.4811	0.4766
C1 colon		1.0000	0.7717	0.5780	0.8443	0.7495	0.6291		0.7879	0.5468	0.4521	0.4602	0.4132	0.4196
C1 pancreas			1.0000	0.6350	0.7323	0.7055	0.5670			0.7920	0.5238	0.4423	0.4525	0.4471
C1 prostate				1.0000	0.6141	0.6128	0.7809				0.8030	0.6705	0.6532	0.5749
C1 breast					1.0000	0.7171	0.6178					0.5597	0.4847	0.4340
C1 lung						1.0000	0.6412						0.5973	0.5432
C1 brain							1.0000							0.7489

C2 stomach	0.7413	0.5065	0.4159	0.4522	0.5590	0.6248	0.4874	1.0000	0.6697	0.6910	0.5982	0.5028	0.5701	0.5574
C2 colon		0.7879	0.6504	0.5070	0.6082	0.7069	0.4928		1.0000	0.6640	0.5867	0.6153	0.6117	0.5969
C2 pancreas			0.7920	0.5260	0.5948	0.5619	0.4985			1.0000	0.6639	0.5507	0.5470	0.5877
C2 prostate				0.8030	0.4666	0.5137	0.6066				1.0000	0.7289	0.6994	0.7280
C2 breast					0.5597	0.4873	0.5653					1.0000	0.7115	0.6674
C2 lung						0.5973	0.6224						1.0000	0.6877
C2 brain							0.7489							1.0000

**Table 13.** Comparison of perturbation results (target rankings) using Spearman’s rank correlation coefficient based on the difference in betweenness method. Only the common targets are compared, and the ranks are relative to this target set.

Network	C1 stomach	C1 colon	C1 pancreas	C1 prostate	C1 breast	C1 lung	C1 brain	C2 stomach	C2 colon	C2 pancreas	C2 prostate	C2 breast	C2 lung	C2 brain
C1 stomach	1.0000	0.1459	0.2992	−0.0061	0.2170	0.3757	0.2768	0.5961	0.1163	0.1800	−0.0062	0.0632	0.3786	0.1459
C1 colon		1.0000	0.0966	0.2042	0.3664	0.4210	0.1141		0.6374	0.0973	0.0895	0.1848	0.2884	−0.0890
C1 pancreas			1.0000	0.1826	0.1697	0.4432	0.1726			0.6436	−0.0030	−0.0652	0.4453	0.0826
C1 prostate				1.0000	0.3136	0.3141	0.1260				0.4012	0.2257	0.2728	0.0600
C1 breast					1.0000	0.2517	0.0244					0.5850	0.1557	–0.0561
C1 lung						1.0000	0.1919						0.6186	0.1034
C1 brain							1.0000							0.6197

C2 stomach	0.5961	0.1158	0.1911	–0.0195	0.1955	0.2902	0.3044	1.0000	0.0931	0.1299	0.0104	0.1024	0.3671	0.1901
C2 colon		0.6374	0.0989	0.1904	0.3064	0.3521	0.0762		1.0000	0.1197	0.1559	0.1884	0.3289	0.0596
C2 pancreas			0.6436	0.1459	0.1149	0.4359	0.1883			1.0000	0.0768	0.0576	0.4307	0.1453
C2 prostate				0.4012	0.0950	0.1934	0.1007				1.0000	0.1627	0.2809	0.1058
C2 breast					0.5850	0.2681	0.1731					1.0000	0.3031	0.1051
C2 lung						0.6186	0.1655						1.0000	0.1340
C2 brain							0.6197							1.0000

**Table 14.** Ratio of average degree for targets with associations over the average degree of all targets in the underlying data source combinations.

Data source combination	Cancer associations	Disease associations	Drug associations
C1	2.2715	1.7777	1.6333
C2	1.9041	1.4378	1.3457

**Table 15.** Ratio of average degree for targets with associations over the average degree of all targets in different cancer networks generated from the curated only data source combination.

Cancer type	Cancer associations	Disease associations	Drug associations
C1 stomach	2.0340	2.0067	2.4387
C1 colon	1.4333	1.3889	1.3356
C1 pancreas	1.8839	1.9936	1.9390
C1 prostate	1.7860	1.7628	2.4421
C1 breast	1.6318	1.5601	1.2491
C1 lung	1.6719	1.9992	1.5839
C1 brain	2.1083	2.1777	1.7906
C2 stomach	1.4832	1.3556	1.6414
C2 colon	0.9918	1.0728	0.9576
C2 pancreas	1.5766	1.4142	1.3132
C2 prostate	1.3732	1.2276	1.6521
C2 breast	1.3467	1.1052	0.9614
C2 lung	1.2333	1.2384	0.9642
C2 brain	1.4802	1.4996	1.5017

**Table 16.** Ratio of average degree for targets with associations over the average degree of all targets in the high-throughput dataset.

Cancer associations	Disease associations	Drug associations
1.2255	1.2793	2.1912

**Table 17.** Ratio of average degree for targets with associations over the average degree of all targets in different cancer networks generated from the high throughput dataset.

Cancer type	Cancer associations	Disease associations	Drug associations
stomach	0.9520	1.0098	1.8037
colon	0.9951	1.3780	2.2372
pancreas	1.0253	0.8898	2.9431
prostate	1.0267	1.3193	2.0682
breast	1.1524	1.1267	1.8870
lung	1.2070	1.2825	2.7589
brain	1.2587	1.1293	2.4459

## Figures and Tables

**Table 1.** CGAP SAGE library IDs used in expression comparisons for each cancer type.

Cancer Type	Cancer	Normal
Stomach	GSM2385	GSM784
Colon	GSM755	GSM728
Pancreas	GSM743	GSM716
Prostate	GSM740	GSM739
Breast	GSM672	GSM14756
Lung	GSM14806	GSM14805
Brain	GSM14762	GSM763

**Table 2.** Data statistics: Topological properties of data sources and data combinations where C1 = BIND + HPRD + DIP and C2 = BIND + HPRD + DIP + Predicted.

	BIND	HPRD	DIP	Predicted	Combination C1	Combination C2
Number of vertices, *N*	2501	3349	597	2696	4524	5989
Number of edges, *n*	3574	5521	730	6958	9020	15776
Average degree, *k*	2.858	3.297	2.446	5.162	3.988	5.268
Average shortest path, *l*	5.300	5.945	6.737	6.485	5.043	5.091
Clustering coefficient, *C*	0.073	0.058	0.094	0.139	0.078	0.104
Assortativity coefficient, *r*	−0.169	0.041	0.031	0.446	–0.111	–0.052

**Table 3.** Topological parameters of selected networks, where the network name reflects the data combination (C1 or C2), cancer type, and whether the network is a cancer or normal network.

Network	Number of vertices, *N*	Number of edges, *n*	Giant component size, *G*	Assortativity coefficient, *r*
C1 stomach cancer	887	586	414	−0.1499
C1 stomach normal	848	501	366	−0.1843
C1 colon cancer	992	709	502	−0.1325
C1 colon normal	1069	843	569	−0.1197
C1 pancreas cancer	898	585	391	0.0584
C1 pancreas normal	859	452	286	0.0106

Network	Average degree, k	Average shortest path, *l*	Clustering coefficient, *C*	Modularity, *Q*
C1 stomach cancer	1.3213	5.8250	0.0309	0.7666
C1 stomach normal	1.1816	5.7118	0.0271	0.7620
C1 colon cancer	1.4294	5.5504	0.0285	0.7332
C1 colon normal	1.5771	5.6176	0.0343	0.7477
C1 pancreas cancer	1.3028	6.8627	0.0335	0.7837
C1 pancreas normal	1.0523	6.7659	0.0299	0.7806

**Table 4.** Clustering coefficients of example cancer and normal networks, compared to equivalent ER and BA model networks with the same *N* and *k*.

Cancer network	ER model	BA model	Observed
C2 stomach cancer	0.0019	0.0019	0.0869
C2 stomach normal	0.0017	0.0018	0.0789
C2 colon cancer	0.0017	0.0020	0.0858
C2 colon normal	0.0016	0.0026	0.0924
C2 pancreas cancer	0.0017	0.0019	0.0901
C2 pancreas normal	0.0020	0.0021	0.1017
C2 prostate cancer	0.0014	0.0072	0.0953
C2 prostate normal	0.0014	0.0077	0.0946
C2 breast cancer	0.0015	0.0047	0.0935
C2 breast normal	0.0016	0.0054	0.0885
C2 lung cancer	0.0016	0.0027	0.0905
C2 lung normal	0.0018	0.0056	0.0921
C2 brain cancer	0.0014	0.0056	0.0940
C2 brain normal	0.0013	0.0040	0.0861

**Table 5.** Average maximum preferential perturbation score for alternative vertex ranking methods and the standard deviation.

Ranking method	Score	σ
Betweenness	0.5589	0.0952
Diffierence in betweenness	0.3910	0.1619
Degree	0.3133	0.1079
Diffierence in degree	0.2412	0.0756
Perturbation scores	0.3691	0.1464

**Table 6.** Average perturbation gradient for alternative vertex ranking methods and the standard deviation.

Ranking method	Gradient	σ
Betweenness	9.9506	2.5663
Diffierence in betweenness	3.9463	2.4213
Degree	3.2155	1.4297
Diffierence in degree	0.7879	0.6310
Perturbation scores	2.7634	2.2960

**Table 7.** Successive targets obtained using alternative ranking methods and the associated perturbation score for the preferential perturbation of prostate networks of data combination type C1.

Vertices	betweenness		diff in betweenness		degree		difference in degree		perturbation scores	

removed	target	score	target	score	target	score	target	score	target	score
1	PXN	−0.0026	SERPINA3	0.0019	PXN	−0.0027	SERPINA3	0.0019	APP	0.0117
2	VCL	−0.0011	APP	0.0136	VCL	−0.0011	SLC9A3R1	0.0038	CDC42	0.0187
3	SMAD2	−0.0006	SLC9A3R1	0.0155	SMAD2	−0.0006	HIST2H2BE	0.0067	MAPK3	0.0216
4	TLN1	−0.0020	DSP	0.0165	TLN1	−0.0020	DSP	0.0077	CDKN1A	0.0246
5	EGFR	−0.0026	HIST2H2BE	0.0193	SMAD3	−0.0036	CBX4	0.0086	PBX2	0.0275

**Table 8.** Linear regression correlation coefficients for initial vertex betweenness between the underlying networks (C1 and C2) and cancer networks.

	C1	C2
C1 stomach	0.8231	0.8080
C1 colon	0.8397	0.8484
C1 pancreas	0.6851	0.6043
C1 prostate	0.8879	0.8274
C1 breast	0.8648	0.8467
C1 lung	0.7963	0.7515
C1 brain	0.8966	0.8545

C2 stomach	0.7625	0.8539
C2 colon	0.7871	0.8441
C2 pancreas	0.6349	0.7356
C2 prostate	0.8211	0.8871
C2 breast	0.8047	0.8701
C2 lung	0.7626	0.8246
C2 brain	0.8245	0.8854

**Table 9.** Average Spearman’s rank correlation coefficient between perturbation results for each vertex ranking method, averaged across all cancer and normal network pairs.

Ranking method	<*r*_*s*_>
betweenness	0.6019
diffierence in betweenness	0.1784
degree	0.6261
diffierence in degree	0.3189
perturbation scores	0.1809

**Table 10.** Combined p-values for alternative ranking methods and target associations. Methods: be-betweenness, db-difference in betweenness, de-degree, dd-diffierence in degree, ps-perturbation scores.

Method	Cancer	Disease	Drug
be	2.47 × 10^−9^	< 1 × 10^−15^	0.0002
db	0.0010	0.0008	0.7168
de	1.92 × 10^−6^	1.73 × 10^−14^	0.0080
dd	0.7260	0.5151	0.7800
ps	1.45 × 10^−5^	1.38 × 10^−13^	0.0006

**Table 11.** Ratio of average betweenness for targets with associations over the average betweenness of all targets in the underlying data source combinations.

Data source combination	Cancer associations	Disease associations	Drug associations
C1	3.4734	2.4497	2.2374
C2	3.4356	2.3218	2.1695

**Table 12.** Ratio of average betweenness for targets with associations over the average betweenness of all targets in different cancer networks for data combination C1.

Cancer type	Cancer associations	Disease associations	Drug associations
stomach	3.7041	3.1512	3.5090
colon	1.5189	1.7063	1.4188
pancreas	2.1791	2.8405	3.0614
prostate	2.6549	2.1653	3.5936
breast	2.3021	1.7587	1.8214
lung	1.9976	2.5478	1.7136
brain	3.5784	3.1194	1.9577

**Table 13.** Ratio of average betweenness for targets with associations over the average betweenness of all targets in the high-throughput dataset.

Cancer associations	Disease associations	Drug associations
1.7238	1.1538	1.8700

**Table 14.** Ratio of average betweenness for targets with associations over the average betweenness of all targets in different cancer networks generated from the high-throughput dataset.

Cancer type	Cancer associations	Disease associations	Drug associations
stomach	1.0841	1.2993	2.5160
colon	1.0573	1.5334	2.2316
pancreas	1.2621	0.8791	2.3329
prostate	0.9520	1.1033	1.8761
breast	1.2542	1.2098	1.9720
lung	1.3149	0.9944	1.9793
brain	1.3337	1.2194	2.4215

**Table 15.** Combined p-values for alternative ranking methods and target associations from perturbations of networks generated from high-throughput data. Methods: be-betweenness, db-difference in betweenness, de-degree, dd-diffierence in degree, ps-perturbation scores.

Method	Cancer	Disease	Drug
be	0.8778	0.1521	2:12 × 10^−5^
db	0.9353	0.7946	0.9955
dd	0.3805	0.5317	0.9453
de	0.6702	0.0694	4.82 × 10^−6^
ps	0.9867	0.9473	0.9937
